# Coupling critical nitrogen dilution curve with hyperspectral feature optimization and machine learning for inversion of nitrogen nutrition index in greenhouse cucumber

**DOI:** 10.3389/fpls.2026.1909263

**Published:** 2026-08-14

**Authors:** Tingting Zhao, Jie Li, Caixia Hu, Donghui Zhang, Guilong Zhang, Yan Xu, Weiming Xiu

**Affiliations:** 1 Agro-Environmental Protection Institute, Ministry of Agriculture and Rural Affairs, Tianjin, China; 2 Aerospace Information Research Institute, Chinese Academy of Sciences, Beijing, China

**Keywords:** greenhouse cucumber, hyperspectral sensing, machine learning, nitrogen nutrition index, precision nitrogen management

## Abstract

**Introduction:**

Rapid, non-destructive, and accurate diagnosis of nitrogen (N) nutritional status in greenhouse cucumber production is critical to mitigate over-fertilization risks and support sustainable intensification.

**Methods:**

An integrated diagnostic framework was developed by coupling the agronomic critical nitrogen (Nc) dilution curve with a hybrid Competitive Adaptive Reweighted Sampling and Variance Inflation Factor (CARS-VIF) feature selection algorithm, followed by benchmarking six machine learning regression models, including K-Nearest Neighbors (KNN), Multilayer Perceptron (MLP), Support Vector Regression (SVR), Extra Trees, Random Forest (RF), and Gradient Boosting Regression (GBR) .

**Results:**

The critical nitrogen dilution curve was parameterized as Nc = 4.378 × DW-0.115 (R² = 0.745), with empirical Nitrogen Nutrition Index (NNI) values ranging from 0.72 to 1.22. The CARS-VIF pipeline compressed the full spectrum down to four sensitive wavebands (430, 677, 688, and 953 nm), achieving a 99.81% dimensionality reduction. GBR emerged as the optimal inversion model, delivering a validation R² of 0.845, RMSE of 0.061, and the lowest MAE of 0.046.

**Discussion:**

This non-destructive framework holds promise for future integration into automated greenhouse sensor platforms or unmanned aerial vehicles (UAVs), offering a powerful digital toolkit to support precision fertilization decision-making and sustainable smart facility horticulture. Additional validation under field conditions is required before operational deployment.

## 1 Introduction

As one of the most economically vital and high-yielding vegetable crops globally, the greenhouse cucumber (*Cucumis sativus* L.) demands intensive nutrient management to sustain its rapid vegetative extension and continuous fruit development (Zou et al., 2011; Sabzi et al., 2021). Within modern protected horticulture, nitrogen (N) stands as the primary macronutrient governing leaf-tissue chlorophyll synthesis, photosynthetic enzyme capacity, and overall dry matter partitioning (Chen et al., 2025). However, driven by the persistent pursuit of maximum financial returns, commercial greenhouse cultivation systems are globally compromised by unscientific, excessive chemical nitrogen inputs. Growers routinely apply fertilizer rates that far exceed the actual biological requirements of the crop, creating a severe structural nutrient imbalance. This chronic over-fertilization does not lead to further crop yield optimization; instead, it triggers cascading ecological crises (Amirahmadi et al., 2024). The massive surplus of unabsorbed nitrogen induces severe agricultural non-point source pollution, characterized by widespread nitrate leaching into shallow groundwater aquifers, secondary soil salinization, and accelerated emissions of nitrous oxide (Geng et al., 2021). To arrest this profound environmental degradation and mitigate agricultural resource waste, the execution of rapid, accurate, and non-destructive nitrogen nutritional status diagnosis has emerged as a paramount scientific prerequisite (Burns et al., 2022). Transitioning from traditional, reactive soil-plant analysis toward proactive, real-time nutrient regulation provides a robust technical path to optimize structural nitrogen investments (Liu et al., 2023). Resolving this diagnostic bottleneck is of profound strategic significance for precision agriculture. It provides a reliable digital decision-making toolkit to balance the trade-offs between high-intensity economic yields and long-term environmental sustainability, directly advancing national and global initiatives for green development and eco-friendly facility horticulture.

To address the critical need for reliable nutrient diagnostics, the historical evolution of agricultural nitrogen tracking has transitioned through three distinct developmental epochs, shifting from destructive macroscopic sampling toward non-destructive microscopic spectral scanning (Zhang et al., 2024). Early agronomic diagnostic systems historically relied upon traditional wet-chemical tissue testing, such as Kjeldahl digestion and micro-analytical laboratory assays (Wang et al., 2018). Although these protocols delivered high absolute chemical accuracy, their practical application in modern facility production was severely constrained by their destructive nature, intensive manual labor, prohibitive logistical costs, and significant temporal delays, which ultimately failed to support real-time greenhouse decision-making (Canicatti et al., 2025). To overcome these temporal lags and provide an objective biological baseline, the second epoch witnessed the integration of mathematical ecological threshold modeling. Scholars systematically introduced the critical nitrogen ( $N_{c}$) dilution curve theory, which is grounded in the functional biological principle that plant nitrogen concentrations decline progressively on a dry-matter basis as structural carbon and architectural shading tissues accumulate (Wang et al., 2023). This mathematical threshold approach successfully enabled the quantitative determination of the Nitrogen Nutrition Index (NNI), providing an immutable physiological indicator to identify genuine nitrogen deficiency ( NNI<1) from excessive luxury consumption ( NNI>1).

Building upon this biological foundation, the third and current developmental epoch has combined these mechanistic dilution frameworks with non-destructive remote sensing technologies to achieve high-frequency monitoring (Padilla et al., 2017). Globally, scientists have deployed advanced leaf-level and canopy-level hyperspectral sensors to capture the subtle optical variations linked to crop tissue nutrient fluctuations. Within this context, extensive agricultural research has emphasized that optimizing alternative nutrient paradigms can substantially mitigate environmental risks without sacrificing crop performance, as elegantly demonstrated by recent top-tier breakthroughs (Geng et al., 2021). Concurrently, comprehensive field benchmarking has further highlighted the imperative of balancing spatial fertilizer allocation with localized crop uptake dynamics to minimize residual nutrient losses (Wang et al., 2019). Modern hyperspectral research has thus moved beyond simple linear vegetation indices, focusing instead on mining the continuous full spectrum (Kovács et al., 2025). By analyzing subtle shifts in light scattering across the blue chlorophyll absorption zone and the near-infrared structural plateau, remote sensing has successfully unlocked the capacity to dynamically track agricultural nutrient distributions under diverse cultivation conditions.

However, successfully bridging the gap between high-dimensional hyperspectral reflectance arrays and mechanistic biological threshold indexes remains a severe scientific challenge (Chang et al., 2024). Despite the progress achieved by marrying optical sensors with machine learning algorithms, two profound technical bottlenecks continue to limit large-scale commercial deployment in protected agriculture. First, full-spectrum continuous hyperspectral datasets are inherently plagued by massive data redundancy and intense, multi-band multicollinearity (Ata-Ul-Karim et al., 2014). The hundreds of contiguous wavebands generate high levels of mathematical noise and irrelevant background variations, which frequently destabilize standard regression equations, inflate computational workloads, and trigger severe overfitting traps during calibration. Second, the mathematical mappings connecting high-dimensional leaf reflectance vectors to crop NNI labels are highly non-linear, context-dependent, and dynamically altered across changing phenological phases (Cannizzaro et al., 2023). Traditional empirical models and simple linear indices routinely fail to isolate genuine physiological nitrogen deficiency stress from complex environmental background noise and canopy architectural variations, thereby compromising model transferability across diverse greenhouse cultivation seasons and distinct management systems.

To systematically resolve these interconnected bottlenecks, this study engineered a robust NNI inversion framework for greenhouse cucumber by coupling agronomic critical nitrogen paradigms with advanced machine learning regression algorithms (Hua et al., 2022; Aakko-Saksa et al., 2023). The experimental dataset encompassed eight distinct cucumber growth stages (S1–S8) and four nitrogen application gradients (CK, N1, N2, and N3), providing a comprehensive basis for model calibration and validation. To overcome spectral data redundancy and severe multicollinearity, a hybrid feature engineering strategy combining Competitive Adaptive Reweighted Sampling with Variance Inflation Factor diagnostics (CARS–VIF) was deployed (Hammer et al., 1999; Mailankody et al., 2022). Six mainstream machine learning regression algorithms, including K-Nearest Neighbors (KNN), Multilayer Perceptron (MLP), Support Vector Regression (SVR), Extra Trees, Random Forest (RF), and Gradient Boosting Regression (GBR), were trained and rigorously evaluated to identify the most robust model for NNI prediction (Yamashita et al., 2020; Feng et al., 2023; Pan et al., 2025). The primary objectives of this study were to: (1) parameterize the critical nitrogen dilution curve for greenhouse cucumber to establish a biologically validated NNI ground-truth; (2) develop an effective feature selection strategy to isolate the most informative wavebands from the full hyperspectral spectrum; and (3) systematically benchmark multiple machine learning algorithms to determine the optimal inversion model for non-destructive nitrogen status diagnosis.

## 2 Materials and methods

### 2.1 Study area

**Figure 1:**
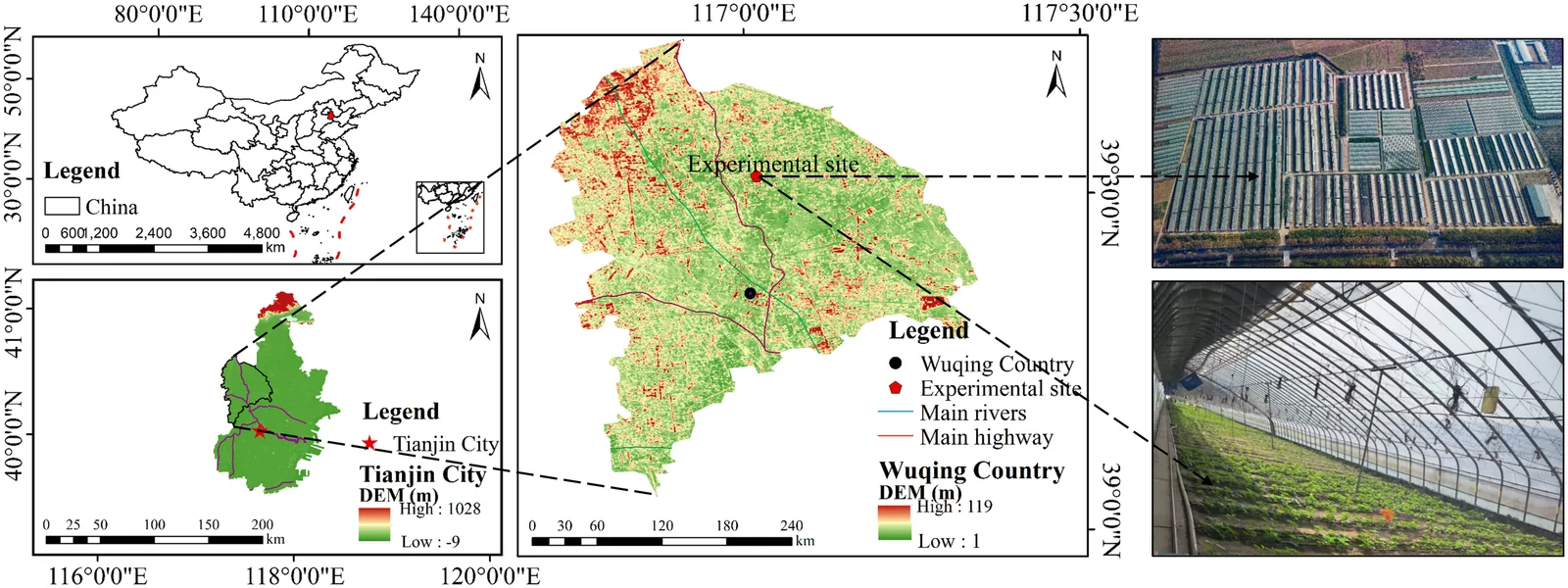
Location of the experimental site. The schematic map illustrates the geographic coordinates and spatial position of the Tianmin Fruit and Vegetable Cooperative within Wuqing District, Tianjin, China.

The experiment was conducted at the Tianmin Fruit and Vegetable Cooperative in Daliang Town, Wuqing District, Tianjin, China (117°2′46″E, 39°32′5″N), a representative protected vegetable production area within the Beijing–Tianjin metropolitan region (**Figure 1**). The study area is characterized by a temperate monsoon climate, with mean annual precipitation of 532 mm, mean annual temperature of 13.5 °C, and annual sunshine duration of approximately 2392 h (Shi et al., 2022). The experiment was carried out in a typical solar greenhouse covered with polyethylene film, which represents the common greenhouse cultivation system used for cucumber production in northern China. The soil was classified as fluvo-aquic soil with sandy loam texture (Yu et al., 2023). The basic physicochemical properties of the 0–20 cm cultivated soil layer were as follows: pH 7.33, Electrical Conductivity (EC) 0.24 mS·cm^-1^, sand 45.0%, silt 39.4%, clay 15.7%, Soil Organic Carbon (SOC) 9.60 g·kg^-1^, Soil Organic Matter (SOM) 16.72 g·kg^-1^, and Total Nitrogen (TN) 1.08 g·kg^-1^. We acknowledge that initial soil nitrate-N, ammonium-N, available phosphorus, and available potassium were not measured at the time of this experiment; however, phosphorus and potassium fertilizers were uniformly applied across all treatments according to local high-yielding guidelines (see Section 2.3.1), which helps ensure that the observed treatment differences were primarily attributable to the nitrogen gradients rather than to limitations of other nutrients.

Characterized by high land re-utilization and intensive chemical inputs, this region serves as a critical sentinel zone for agricultural resource optimization (Lu et al., 2024). Furthermore, implementing a machine-learning-driven nitrogen diagnostic framework here carries profound eco-economic implications. Methodologically, it shifts the local management paradigm from traditional empirical over-fertilization to real-time, non-destructive precision management (Pourdarbani et al., 2020). Consequently, this baseline framework not only dramatically curtails fertilizer waste and mitigates groundwater nitrate leaching risks, but also provides a scalable blueprint for sustainable, high-efficiency green vegetable production in intensive facility conglomerates globally.

### 2.2 Comprehensive methodological framework

**Figure 2:**
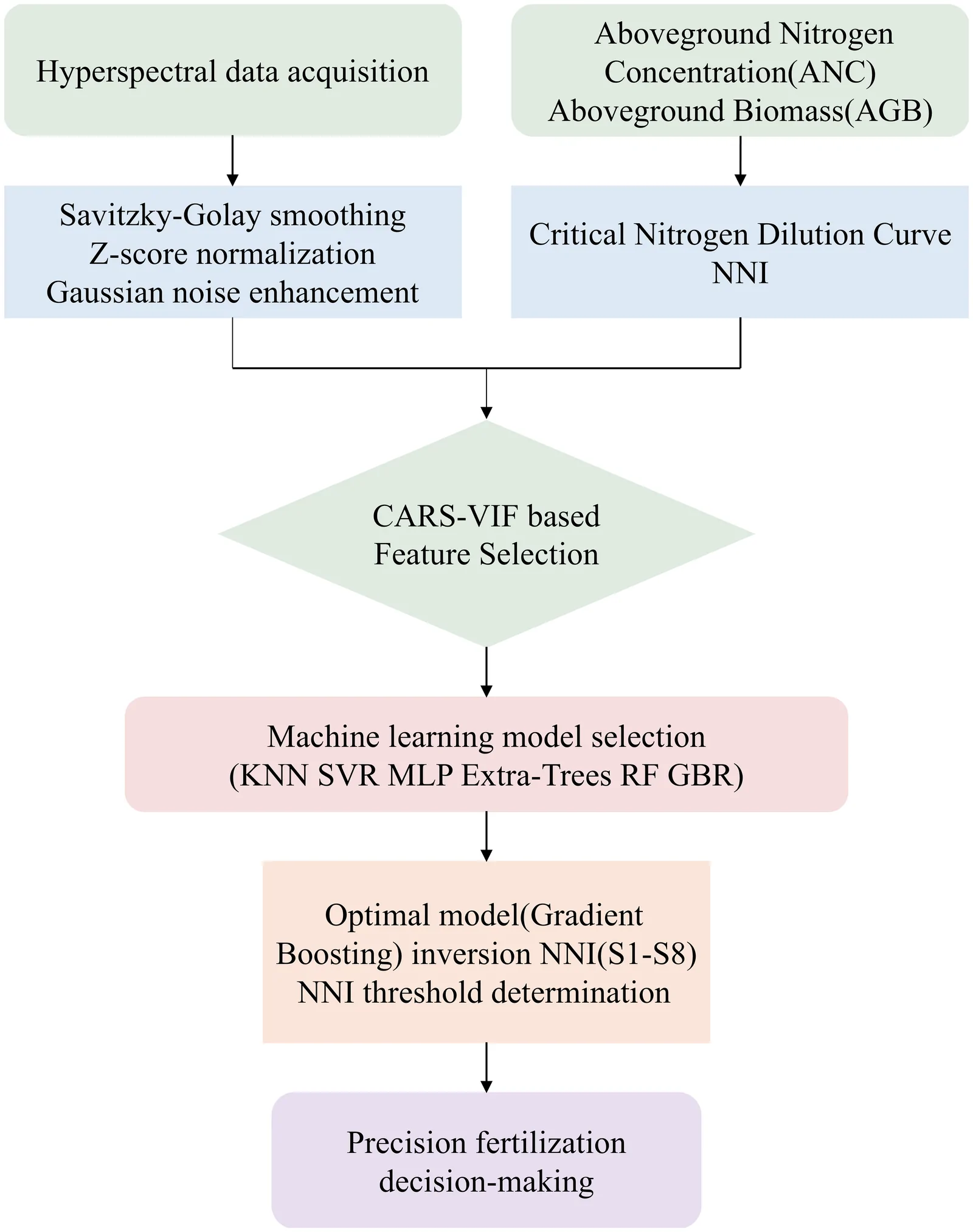
Comprehensive workflow of the study. The analytical procedure comprised the following sequential steps: (1) greenhouse cucumber experiments with four nitrogen treatments across eight growth stages; (2) leaf hyperspectral reflectance acquisition (nine points per leaf, five leaves per plot, three replicate plots) and destructive biomass sampling; (3) organ-level dry mass and nitrogen concentration measurement, followed by aboveground biomass (AGB) and aboveground nitrogen concentration (ANC) calculation, and critical N dilution curve parameterization to derive the empirical Nitrogen Nutrition Index (NNI); (4) CARS-VIF feature selection performed exclusively on the training set using original measured spectra; (5) data augmentation applied only to the training set after the 8:2 split; (6) training and evaluation of six machine learning regression models; and (7) independent testing on the untouched test set to assess model generalization performance.

The systematic implementation workflow developed for diagnosing and inverting the nitrogen nutritional status of greenhouse cucumber is schematically illustrated in **Figure 2**. Structurally encapsulating the methodology, field plots were initially established under four distinct nitrogen application gradients across eight representative growth stages to acquire leaf hyperspectral reflectance datasets alongside synchronized agronomic traits. Subsequently, these biophysical parameters were utilized to construct the critical nitrogen dilution curve, allowing the quantitative calculation of the NNI as the ground-truth modeling target (Canicatti et al., 2025). To eliminate the redundant noise inherent in full-spectrum data, a hybrid feature engineering strategy combining Competitive Adaptive Reweighted Sampling (CARS) and Variance Inflation Factor (VIF) analysis was deployed to isolate sensitive multi-band features (Geremew et al., 2023). Simultaneously, six mainstream machine learning regression models were trained and rigorously validated using a partitioned training-testing dataset framework. This comprehensive pipeline systematically evaluated and identified the most robust mathematical model for non-destructive precision nitrogen management.

### 2.3 Data collection

#### 2.3.1 Experimental layout and nitrogen treatments

The agronomic experiment was arranged in a completely randomized block design with three replications, comprising four distinct nitrogen (N) fertilization gradients: CK (0 kg·ha^-1^), N1 (240 kg·ha^-1^), N2 (480 kg·ha^-1^, local conventional rate), and N3 (720 kg·ha^-1^). Each experimental plot occupied an area of 33 m^2^, isolated by plastic film barriers to prevent lateral nutrient and water movement. Cucumber seedlings (cv. ‘Jinyou 35’) were transplanted into rows with a uniform spacing. Nitrogen fertilizer, applied as urea, was split across six critical growth stages (seedling, flowering, fruit-setting, early, peak, and late fruit-bearing stages) to synchronize with crop nutrient demands (Sun et al., 2012). Other macronutrients, including phosphorus (P_2_O_5_) and potassium (K_2_O), were uniformly managed across all treatments according to local high-yielding guidelines (**Table 1**).

**Table 1:** Nitrogen fertilizer application rates (kg·ha^-1^) at different growth stages.

Treatments	Stage					
	Seedling	Flowering	Fruit-setting	Early fruit-bearing	Peak fruit-bearing	Late fruit-bearing
N1	12	24	12	60	108	24
N2	24	48	24	120	216	48
N3	36	72	36	180	324	72

CK indicates the no-N control. N1, N2, and N3 represent low, conventional, and high N application treatments, respectively. The values in the table indicate the amount of N applied at each growth stage; N at the seedling stage was applied as basal fertilizer, whereas N at the other stages was applied as topdressing.

**Figure 3:**
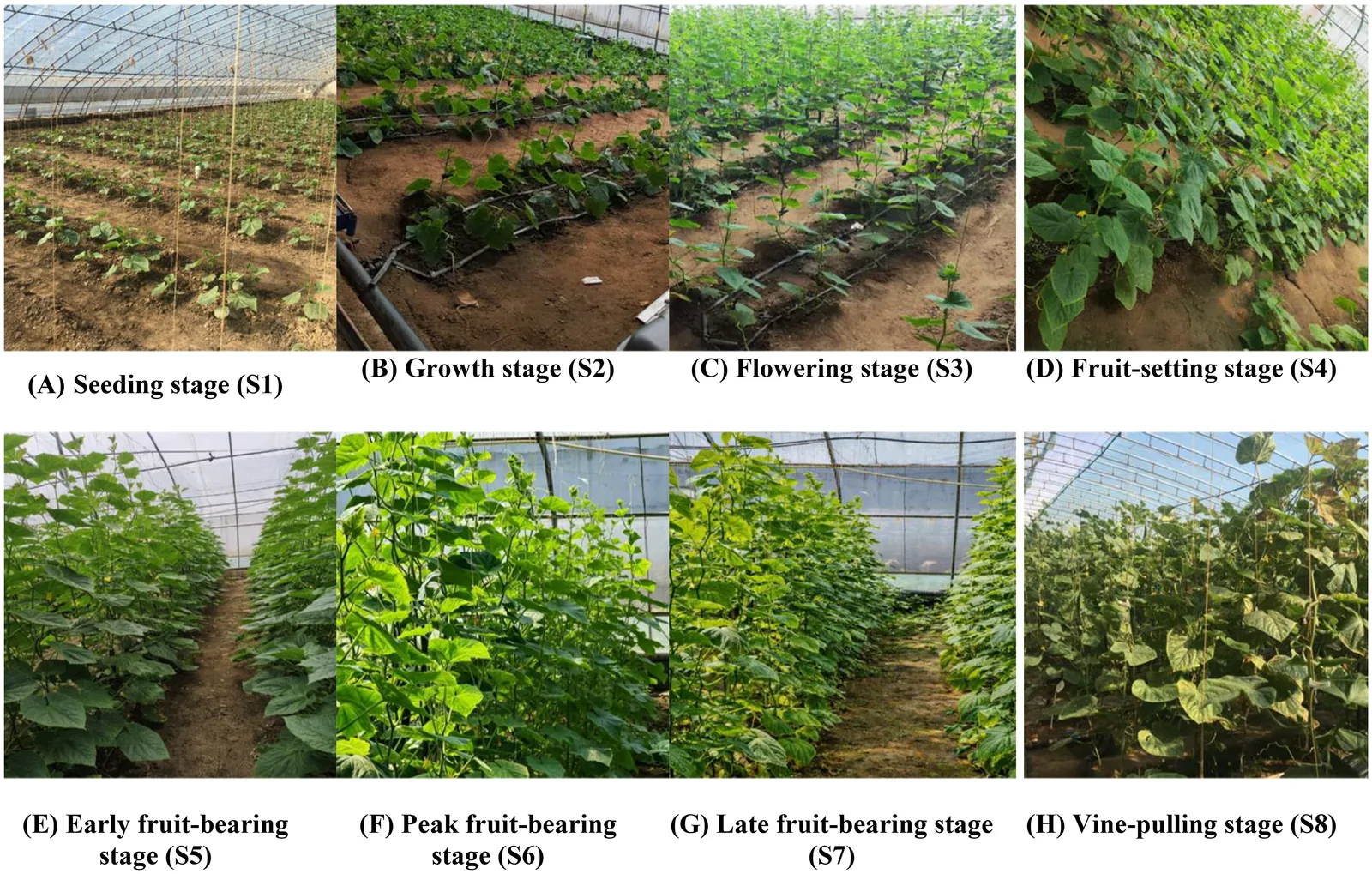
Temporal dynamics of phenotypic development in greenhouse cucumber across eight critical growth stages (S1–S8). S1, seedling stage; S2, vegetative growth stage; S3, flowering stage; S4, fruit-setting stage; S5, early fruit-bearing stage; S6, peak fruit-bearing stage; S7, late fruit-bearing stage; S8, vine-pulling stage. Panels **(a–h)** correspond to S1–S8, respectively.

Data were collected at eight growth stages during the cucumber growing period, corresponding to 20, 30, 40, 45, 50, 55, 60, and 65 days after transplanting. These sampling dates covered the main developmental stages of greenhouse cucumber, including the seedling stage (S1), vegetative growth stage (S2), flowering stage (S3), fruit-setting stage (S4), early fruit-bearing stage (S5), peak fruit-bearing stage (S6), late fruit-bearing stage (S7), and vine-pulling stage (S8), as shown in **Figure 3** (Shi et al., 2012). At each sampling stage, healthy and representative plants were selected from each treatment and replicate plot, excluding border plants and plants with visible damage, diseases, or pest symptoms. The selected plants were used for subsequent measurements of cucumber growth and phenotypic traits.

#### 2.3.2 Leaf hyperspectral reflectance acquisition

Non-destructive leaf hyperspectral reflectance datasets were captured at eight sequential growth stages (denoted as S1 to S8, covering from seedling to vine-pulling stages). At each sampling stage, five healthy, representative plants were randomly selected from each plot and transferred to the laboratory for precise spectral measurements. From these five plants, three representative individuals were subsequently selected for destructive biomass and nitrogen analysis (see Section 2.3.3). Reflectance data were acquired using an AvaSpec-2048-USB2 fiber-optic spectrometer (Avantes BV, Netherlands) spanning a spectral range of 278–1100 nm (Wang et al., 2022). This spectrometer is engineered with a Czerny–Turner optical configuration and a UA grating of 300 lines mm^-1^. The spectral acquisition system integrated a standard SMA optical fiber, an external halogen light source, and a high-reflectance white reference panel (>99% reflectance). To systematically eliminate dark current perturbations and normalize the raw spectral signal, dark-current and white-reference calibrations were strictly performed before each measurement campaign. The relative leaf reflectance (*R*) was normalized using the following Equation 1:

(1) $$R=100*(\frac{sample_{n}-dark_{n}}{ref_{n}-dark_{n}})$$

where $sample_{n}$ is the sample spectrum, $dark_{n}$ is the dark spectrum, and $ref_{n}$ is the white-reference spectrum.

**Figure 4:**
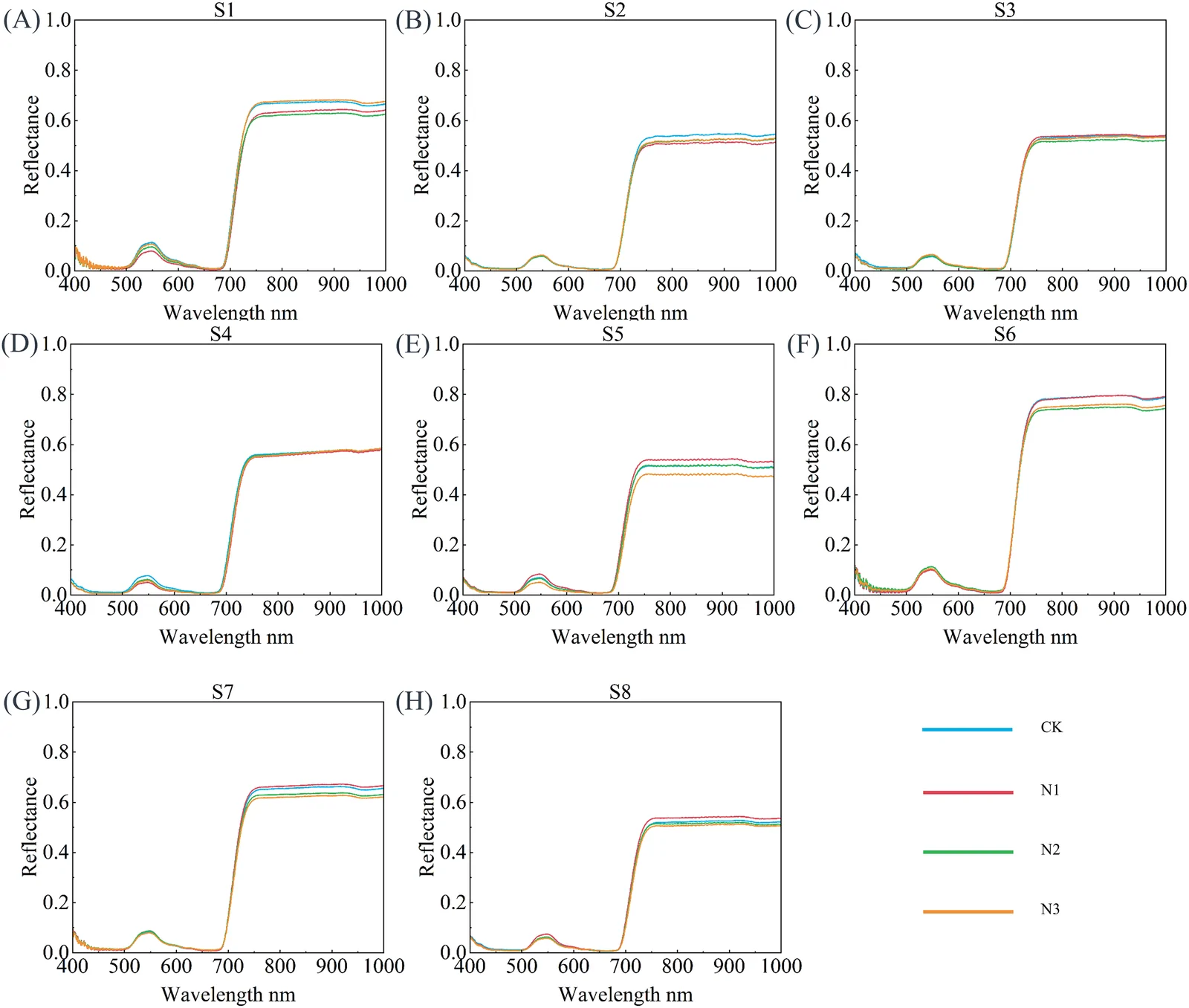
Leaf reflectance spectra of cucumber under different nitrogen treatments across growth stages (S1–S8). Each curve represents the treatment mean (n = 15 leaf spectra per treatment per stage), where each individual spectrum was the average of nine measurement points per leaf. **(A–H)** correspond to growth stages S1–S8, respectively. No error bands are shown as the figure is intended to illustrate treatment-level central tendencies.

**Figure 5:**
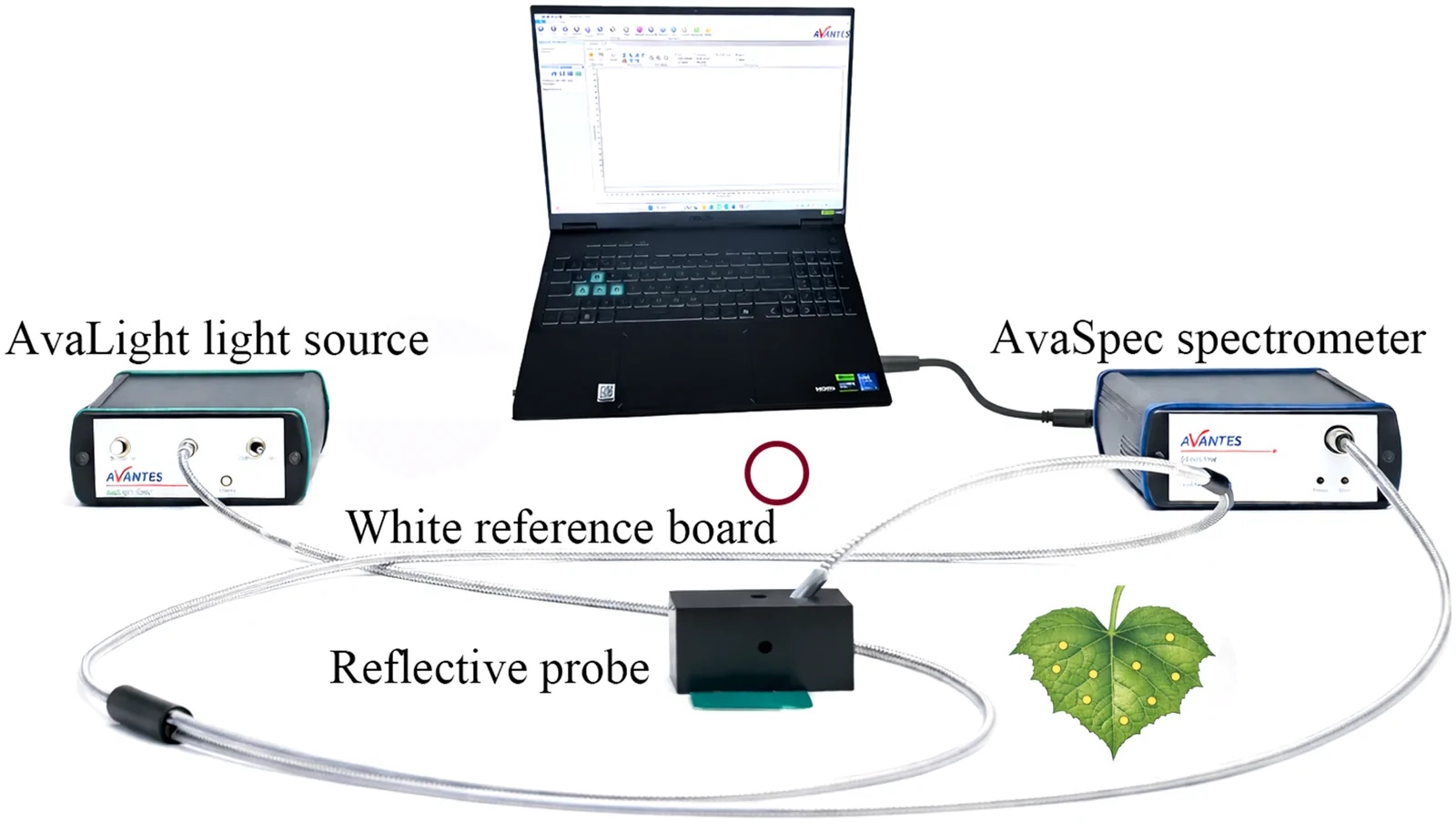
Schematic diagram of cucumber leaf hyperspectral sampling. The system consisted of an AvaLight light source, a reflective fiber-optic probe, a white reference board, and an AvaSpec spectrometer. Nine points were measured on each leaf, avoiding major veins and damaged areas.

During data collection, the fourth to fifth fully expanded functional leaves from the plant top were selected as target samples. To ensure high signal stability and reduce ambient stray light interference, the fiber-optic probe was held strictly perpendicular to the leaf surface. For each leaf sample, nine measurement points were evenly distributed across the leaf blade while carefully avoiding major veins and visibly damaged areas. Ultimately, the average spectrum of these nine points was calculated to serve as the representative spectrum of each individual leaf. For each treatment and growth stage, the final spectral curve was obtained by averaging all individual leaf spectra from the five selected plants across the three replicate plots (i.e., 15 leaf spectra per treatment per stage). These treatment-mean spectral curves are presented in **Figure 4**. No augmented data were used for this figure; all curves are based on original measured spectra (**Figure 5**).

#### 2.3.3 Destructive biomass sampling and plant nitrogen analysis

Immediately following each hyperspectral collection campaign, destructive plant sampling was synchronously performed on the same set of plants. Specifically, from the five plants selected for spectral measurements, three representative individuals were harvested from each plot at every growth stage. The remaining two plants were retained as backup. This ensured that spectral data, biomass, and plant nitrogen concentration were obtained from the same plant population at each sampling stage. The harvested plants were thoroughly rinsed with deionized water. The sampled plants were separated into distinct organs (leaves, stems, and fruits). Each organ was oven-dried at 105 °C for 30 min to deactivate enzymes, and subsequently desiccated at 75 °C until attaining a constant weight to determine the dry mass of each organ (*DM_leaf_*, *DM_stem_*, and *DM_fruit_*). Aboveground biomass (DW, t/hm²) was calculated as the sum of all organ dry masses. After drying, tissue samples from each organ were finely pulverized and sieved through a 0.5-mm mesh (Sun et al., 2023). Plant tissue total nitrogen concentration (PNC, %) was determined separately for each organ using the micro-Kjeldahl digestion method. The aboveground nitrogen concentration (ANC, %) was then calculated as the dry-mass-weighted average of organ nitrogen concentrations (Equation 2):

(2) $$ANC=\frac{DM_{leaf}\times N_{leaf}+DM_{stem}\times N_{stem}+DM_{fruit}\times N_{fruit}}{DW}$$

where, $DM_{leaf}$, $DM_{stem}$, and $DM_{fruit}$ are the dry masses of leaves, stems, and fruits, respectively; $N_{leaf}$, $N_{stem}$, and $N_{fruit}$ are the corresponding nitrogen concentrations; and DW is total aboveground biomass. These empirical biophysical datasets were subsequently utilized to parameterize the critical nitrogen dilution curve and calculate the empirical nitrogen nutrition index (NNI). For each plot and growth stage, the NNI was calculated as the ratio of the measured ANC to the critical nitrogen concentration derived from the same set of harvested plants.

Plant tissue total nitrogen concentration (PNC, %) was quantitatively determined utilizing the micro-Kjeldahl digestion method. These empirical biophysical datasets were subsequently utilized to parameterize the critical nitrogen dilution curve and calculate the empirical nitrogen nutrition index (NNI). For each plot and growth stage, the NNI was calculated as the ratio of the measured plant nitrogen concentration to the critical nitrogen concentration, both derived from the same set of harvested plants, ensuring direct correspondence between spectral data, biomass, and nitrogen status indicators.

### 2.4 Mathematical modeling and feature engineering

#### 2.4.1 Spectral feature selection for hyperspectral and laboratory test data

To reduce spectral redundancy and improve model robustness, competitive adaptive reweighted sampling (CARS) was used to select informative spectral variables from both hyperspectral and laboratory spectral datasets. CARS is a partial least squares regression (PLSR)-based variable selection method that evaluates wavelength importance according to the absolute values of PLS regression coefficients (Koss et al., 2017). Wavelengths with larger absolute regression coefficients are considered to contribute more strongly to the prediction of the target variable, whereas variables with smaller contributions are progressively eliminated during the iterative selection process.

Let the preprocessed spectral matrix be denoted as (Equation 3):

(3) $$X={\left[x_{ij}\right]}_{n\times p}$$

where n is the number of samples and p is the number of spectral variables.

The response variable is expressed as (Equation 4):

(4) $$y={\left[y_{1},y_{2},\cdots ,y_{n}\right]}^{T}$$

where $y_{i}\;$represents the measured value of the i-th sample, n is the number of samples, and Tdenotes the transpose operation.

Importantly, the entire CARS-VIF feature selection procedure was conducted exclusively on the training set using the original measured spectra (n = 307), not the augmented spectra. The test set (n = 77) was not involved in any stage of feature selection, ensuring that the selected wavebands were identified without access to test set information and preventing information leakage. During each CARS iteration, 80% of the samples were randomly selected by Monte Carlo sampling to establish a local PLSR model (Equation 5):

(5) y=Xβ+ϵ

where X is the spectral matrix, y is the response variable, β is the regression coefficient vector, and ϵ is the residual term.

The importance weight of the j-th wavelength was calculated as (Equation 6):

(6) $$\omega_{j}^{\left(t\right)}=\frac{\left|\beta_{j}^{\left(t\right)}\right|}{\sum_{j=1}^{pt}|\beta_{j}^{\left(t\right)}|}$$

where $\omega_{j}$ is the weight of the j-th wavelength, $\beta_{j}\;$is its regression coefficient, and p is the number of spectral variables.

During the CARS process, variables with smaller regression coefficients were gradually removed using an exponentially decreasing function (Equation 7):

(7) $$r_{t}=\mu e^{-kt}$$

where $r_{t}$ is the proportion of retained variables in the t-th iteration, and μ and k are constants.

For each selected variable subset, a PLSR model was built and evaluated using five-fold cross-validation. The root mean square error of cross-validation (RMSECV) was calculated as (Equation 8):

(8) $$RMSECV=\sqrt{\frac{1}{n}\sum_{i=1}^{n}{\left(y_{i}-{\hat{y}}_{i,cv}\right)}^{2}}$$

where $y_{i}$is the measured value and ${\hat{y}}_{i}$is the predicted value obtained from cross-validation. The variable subset with the lowest RMSECV was regarded as the optimal wavelength subset. To evaluate multicollinearity among the selected wavelengths, the variance inflation factor(VIF)was calculated (Equation 9):

(9) $$VIF_{j}=\frac{1}{1-R_{j}^{2}}$$

where $R_{j}^{2}$is the coefficient of determination obtained by regressing the j-th variable against the remaining selected variables. A higher VIF indicates stronger multicollinearity. The variance inflation factor (VIF) was calculated. Variables with VIF > 10 were considered to have severe multicollinearity.

#### 2.4.2 Critical nitrogen dilution curve and nitrogen nutrition index

The critical nitrogen concentration (%Nc) is defined as the nitrogen concentration required to achieve maximum plant growth. This concept was later developed into the critical nitrogen dilution curve, which describes a power-law decline in critical nitrogen concentration as biomass increases (Colnenne et al., 1998). This model has become an important tool for diagnosing crop nitrogen status and guiding precision nitrogen application.

The procedure used in this study to construct the cucumber critical nitrogen dilution curve was as follows: (1) Aboveground biomass and nitrogen concentration under different nitrogen levels were compared, and nitrogen limitation was determined using ANOVA; (2) For nitrogen-limited treatments, a linear fit between Aboveground Biomass (AGB) and Aboveground Nitrogen Concentration (ANC) was established; (3) For non-limited treatments, the mean biomass was taken as the maximum biomass; (4) The critical nitrogen concentration at each sampling date was defined as the y-coordinate of the intersection of the fitted line from step (2) with the vertical line at maximum biomass. A power function fitted to these critical points across dates yielded the critical nitrogen curve (Equation 10):

(10) $$\%Nc=a*DW^{-b}$$

where %Nc is the critical nitrogen concentration of the aboveground cucumber plant, DW is aboveground dry weight (t·ha^-1^), and a and b are curve parameters. The nitrogen nutrition index (NNI) is the ratio of actual nitrogen concentration to critical nitrogen concentration (Reyes et al., 2016), and it directly reflects the nutritional status of nitrogen in the crop. It can be expressed as (Equation 11):

(11) $$NNI=\frac{Nt}{NC}$$

Where Nt is the measured nitrogen concentration and Nc is the critical nitrogen concentration estimated at the corresponding biomass. *NNI* = 1 indicates optimal nitrogen status, *NNI* > 1 indicates nitrogen surplus, and *NNI* < 1 indicates nitrogen deficiency.

#### 2.4.3 Modeling method

To establish a highly precise and generalized inversion framework for greenhouse cucumber NNI, the core mathematical philosophy relies on systematic benchmarking across multiple advanced regression paradigms to conquer the challenges of high dimensionality, severe multi-band collinearity, and complex biochemical non-linear interactions inherent in leaf hyperspectral datasets. Relying on a single mathematical framework often introduces structural bias and compromises inversion transferability under shifting greenhouse microclimates. Therefore, this study strategically engineered a dual-layered algorithmic exploratory matrix comprising three classic individual mappers and three state-of-the-art ensemble meta-learners to balance structural risk, mathematical flexibility, and computational scaling.

To guarantee mathematical robustness against high-frequency spectral noise and prevent overfitting under typical agronomic small-sample conditions, a Gaussian-noise-driven data augmentation pipeline was deployed. Critically, the augmentation procedure followed the 8:2 randomized data split, rather than preceding it. Specifically, the original dataset (n = 384) was first partitioned into a training set (n = 307) and a test set (n = 77). Augmentation was then applied exclusively to the training set, ensuring that no synthetic sample derived from a training set sample ever appeared in the test set. For each original training sample, three synthetic copies were generated by adding Gaussian noise with a mean of 0 and a standard deviation of 0.01 (in reflectance units), yielding an expanded training set of 1228 samples. The test set remained completely untouched and was retained as fully independent samples to secure transparent, unbiased model evaluations. The noise level was selected based on repeated measurements of a stable white reference standard, where the observed standard deviation across replicate scans ranged from 0.008 to 0.012. To ensure physical plausibility, all augmented reflectance values were constrained to the range of 0–100% reflectance. To ensure the robustness of model comparison, ten repetitions of 5-fold cross-validation were additionally performed on all six final models, and the mean ± standard deviation of R², RMSE, and MAE were calculated to provide statistical confidence in model ranking.

The engineered algorithmic matrix comprehensively exploits divergent statistical learning behaviors to capture the hidden non-linear mappings between target sensitive wavelengths and cucumber NNI. Within the baseline individual mappers, K-Nearest Neighbors (KNN) utilizes instance-based geometric distance matrices to construct non-parametric, local pixel-neighborhood predictions (Ramchandra et al., 2024); Support Vector Regression (SVR) maps input wavebands into a high-dimensional feature space, invoking an ϵ-insensitive loss framework governed by the structural risk minimization principle to secure robust hyperplanes optimized via cross-validated penalty ( C) and kernel ( γ) coefficients (Ghimire et al., 2019); and the Multilayer Perceptron (MLP) employs a hierarchical feedforward neural network structure utilizing error backpropagation through hidden layers to resolve complex multi-neuron non-linear functions (Kashiri et al., 2012). Conversely, the ensemble learners capitalize on bagging and boosting mechanisms to fundamentally elevate prediction ceilings. Random Forest (RF) and Extremely Randomized Trees (Extra Trees) both construct dense random decision forest architectures to decouple feature correlation and minimize variance via statistical averaging, though Extra Trees injects greater structural stochasticity by selecting random cut-points to further eliminate over-fitting traps (Zhang et al., 2025). Lastly, Gradient Boosting Regression (GBR) executes a forward-stage-wise additive expansion, sequentially coupling weak regression tree base learners to minimize custom empirical loss functions, explicitly prioritizing difficult-to-fit spectral residuals to extract latent target signals.

The primary scientific benefit of constructing this multi-paradigm comparative architecture is the execution of a rigorous empirical stress-test across highly divergent learning boundaries, ensuring that the selected optimal inversion framework is mathematically justified rather than arbitrarily assumed. By evaluating models that operate on drastically different mathematical foundations (ranging from instance-based geometry to sequential gradient optimization), this approach successfully captures both localized structural fluctuations and macroscopic non-linear patterns across different cucumber reproductive phases. Methodologically, it shifts the precision nitrogen diagnostic paradigm from rigid, empirical single-index regressions toward a dynamic, data-driven framework capable of isolating genuine physiological stress from spectral background noise. Ultimately, this comprehensive evaluation validates that the chosen machine learning strategy provides a stable, highly transferable, and non-destructive decision-making toolkit for real-time greenhouse cucumber nutrient optimization.

All six machine learning models were implemented using their default hyperparameters as provided in the scikit-learn library (version 1.3.0) to ensure a fair comparison among algorithms. For GBR, the default parameter settings were: n_estimators = 100, learning_rate = 0.1, max_depth = 3, subsample = 1.0, and loss = ‘squared_error’. It should be noted that these default values were used directly without systematic hyperparameter tuning, as our primary objective was model selection (i.e., identifying the most suitable algorithm for NNI prediction) rather than model optimization per se. The default parameters were chosen based on the rationale that they provide a balanced baseline for cross-algorithm comparison.

### 2.5 Model evaluation and data processing

To verify the predictive capability and agronomic stability of the machine learning paradigms for greenhouse cucumber nitrogen tracking, the model performance was comprehensively evaluated using three statistical metrics: the coefficient of determination (*R*^2^), the root mean square error (*RMSE*), and the mean absolute error (*MAE*) (Esmaeili et al., 2024). The philosophy behind this tripartite suite is to balance the assessment of global fitting variance against localized prediction residuals, ensuring the selected model translates into reliable field decision tools.

Specifically, *R*^2^ was deployed to quantify the proportion of total variance in the observed cucumber NNI explained by the leaf hyperspectral inputs, serving as the baseline measure of goodness-of-fit. Concurrently, RMSE was selected due to its sensitivity to larger individual errors, which is critical in agronomic diagnosis because overestimating or underestimating nitrogen could trigger severe fertilizer stress or environmental leaching. Complementing these, MAE was calculated to provide an intuitive measurement of the average expected prediction error across all growth stages. These metrics were mathematically defined as follows (Equations 12–Equation 14):

(12) $$R^{2}=1-\frac{\sum_{i=1}^{n}{\left(y_{i}-{\hat{y}}_{i}\right)}^{2}}{\sum_{i=1}^{n}{\left(y_{i}-\bar{y}\right)}^{2}}$$

(13) $$MAE=\frac{1}{n}\overset{n}{\underset{i=1}{\sum }}|y_{i}-{\hat{y}}_{i}|$$

(14) $$RMSE=\sqrt{\frac{1}{n}\sum_{i=1}^{n}{\left(y_{i}-{\hat{y}}_{i}\right)}^{2}}$$

where $y_{i}$ represents the measured ground-truth NNI of the *i*-th greenhouse cucumber sample, ${\hat{y}}_{i}$ denotes the algorithm-predicted NNI, $\bar{y}$ is the mean of the observed NNI dataset, and *n* signifies the total number of validation samples.

## 3 Results

### 3.1 Dilution curve construction and spatiotemporal dynamics of cucumber NNI

**Figure 6:**
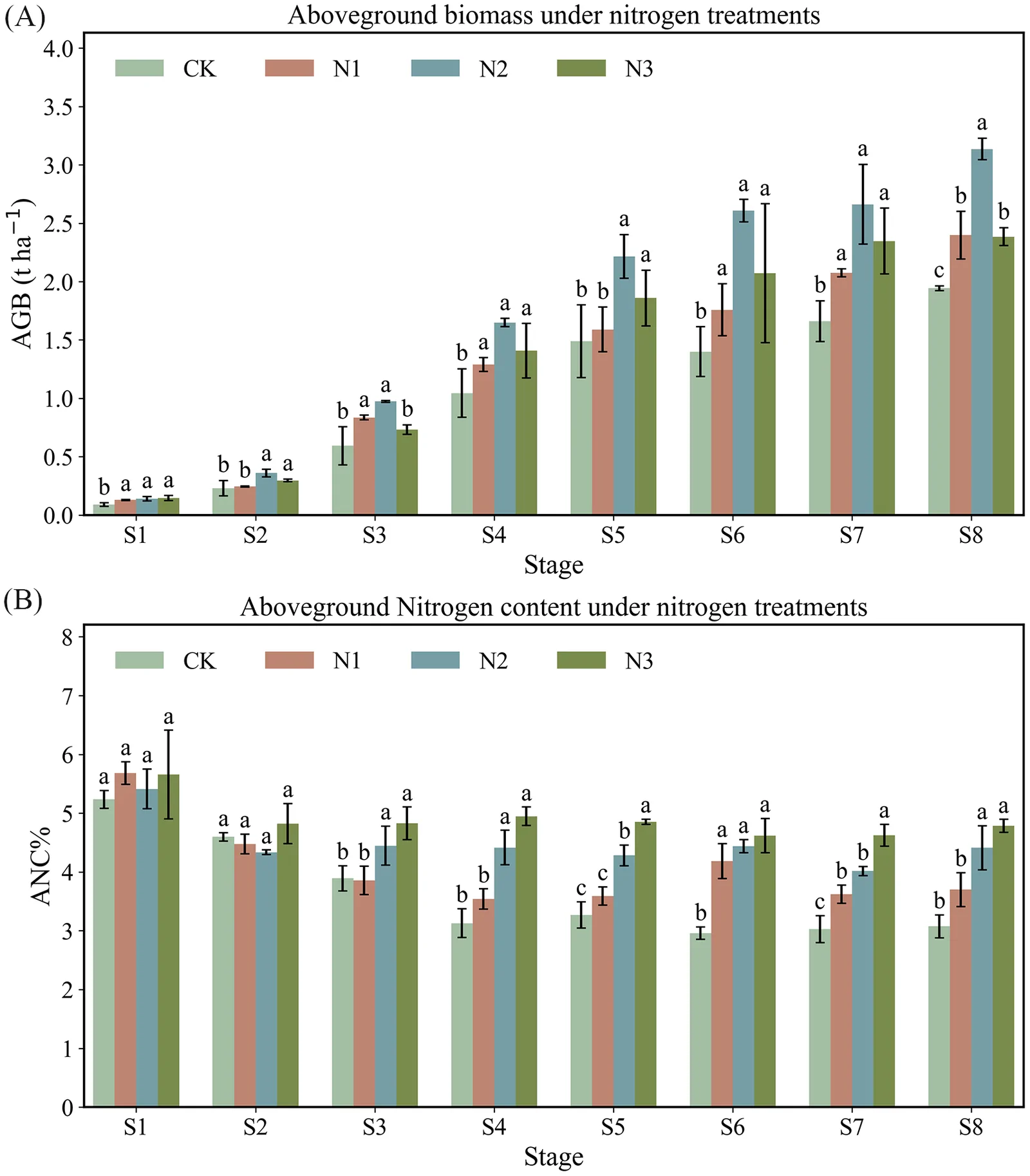
Dynamic changes in aboveground biomass **(A)** and aboveground nitrogen content **(B)** of cucumber plants during the growth stages (S1–S8).

**Figure 7:**
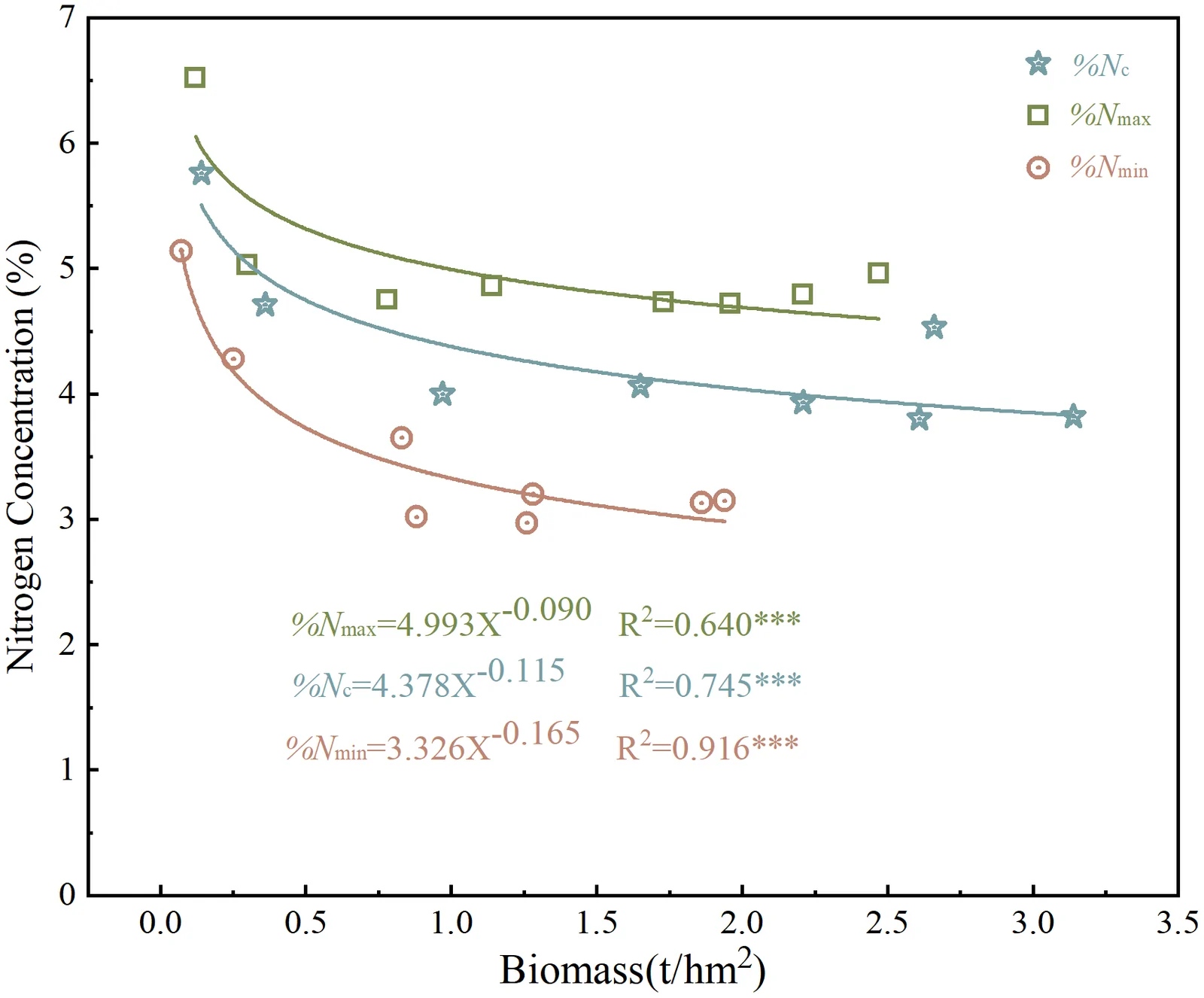
Nitrogen concentration dilution curves of cucumber. %Nc denotes the critical nitrogen concentration dilution curve, while %Nmax and %Nmin indicate the maximum and minimum nitrogen dilution curves, respectively.

Nitrogen treatments significantly affected aboveground biomass (AGB) and aboveground nitrogen concentration (ANC) during cucumber growth (*p* < 0.05). Dynamically, AGB exhibited a steady accumulation, progressing from 0.13–0.14 t/hm² at the seedling stage (S1) to 2.38–3.14 t/hm² at the final harvesting stage (S8), as illustrated in **Figure 6**. From the flowering stage (S3) onward, a pronounced divergence in AGB was observed among the distinct fertilization levels; specifically, the medium-nitrogen (N2) and high-nitrogen (N3) treatments generated significantly higher biomass than the low-nitrogen (N1) and control (CK) plots during both the flowering and fruiting phases. Conversely, ANC displayed a progressive declining trajectory as the crop developed (**Figure 7**). At the S1 stage, leaf-tissue ANC remained relatively high, ranging between 52.35 and 56.57 $\text{g}\cdot {\text{kg}}^{-1}$. Thereafter, dilution took place as structural carbon accumulated. From the S1 stage to the peak fruit-bearing stage (S6), ANC decreased by 43.4%, 25.4%, 17.9%, and 18.3% under the CK, N1, N2, and N3 treatments, respectively, with a substantially steeper decline observed in the nitrogen-deficient groups (CK and N1) compared to the well-fertilized groups.

**Figure 8:**
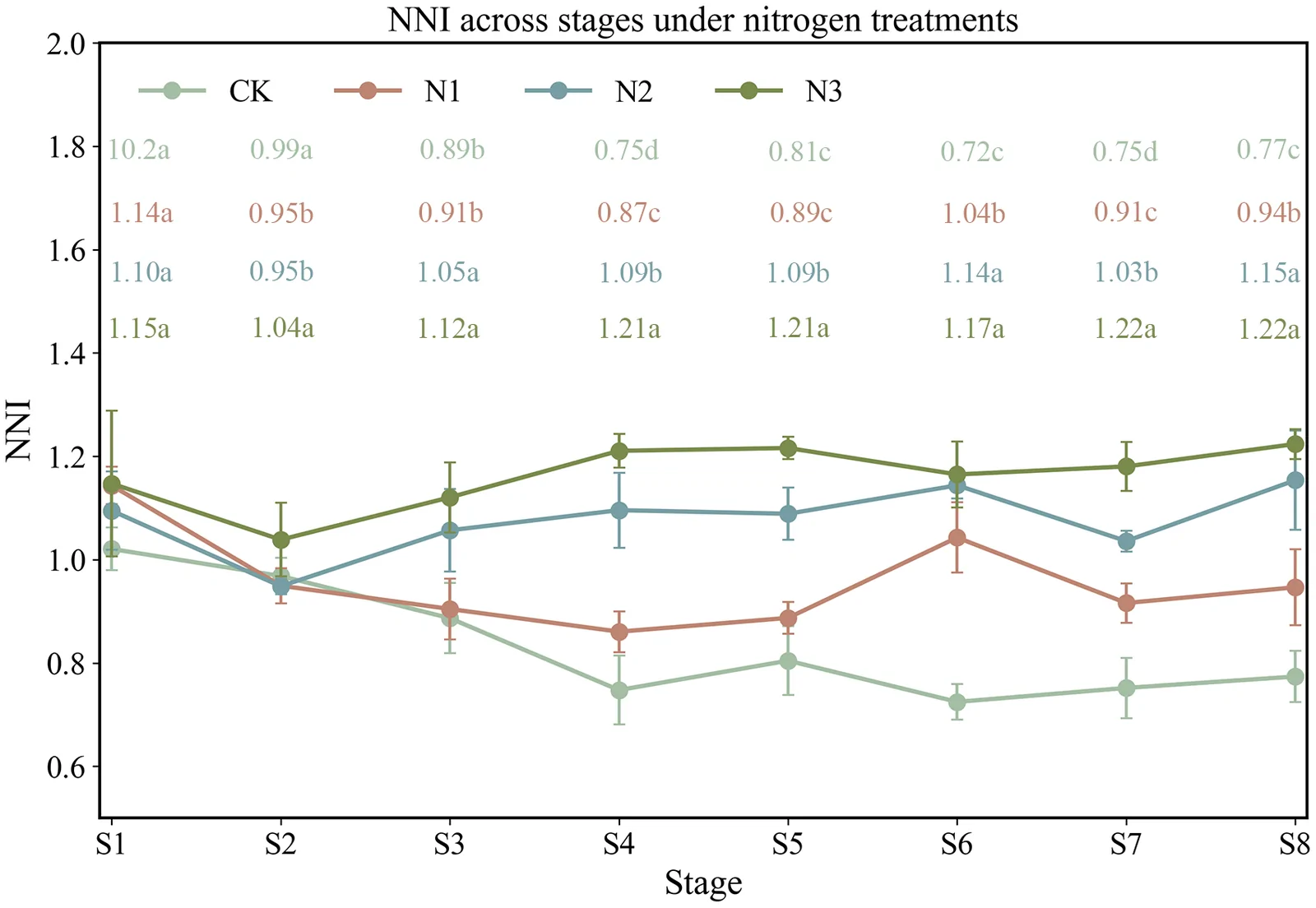
Variation trends of the nitrogen nutrition index (NNI) in cucumber under different nitrogen treatments during growth stages (S1–S8).

Based on the relationship between AGB and ANC, the cucumber critical nitrogen dilution curve was fitted as $N_{c}=4.378\times {\text{DW}}^{-0.115}$, with a coefficient of determination (*R*^2^) of 0.745 ( p<0.001), which is graphically fitted in **Figure 8**. This *R*^2^ value is comparable to the only previously reported cucumber critical N dilution curve under protected cultivation ( $N_{c}=4.539\times {\text{DW}}^{-0.060}$, *R*^2^ = 0.7496), with differences in parameters *a* and *b* attributable to cultivar and environmental variation (Wang et al., 2025). The corresponding boundary upper and lower references governing maximum and minimum nitrogen dilution envelopes were developed as $N_{max}=4.993\times {\text{DW}}^{-0.090}$ (*R*^2^ = 0.640, p<0.001) and $N_{min}=3.326\times {\text{DW}}^{-0.165}$ (*R*^2^ = 0.916, p<0.001), respectively, delineating the rigorous biological thresholds of cucumber nitrogen status.

The empirical Nitrogen Nutrition Index (NNI) values calculated from this baseline framework ranged from 0.72 to 1.22 across growth stages, revealing distinct, statistically significant fluctuations among nitrogen treatment groups ( p<0.05), as dynamically monitored in **Figure 8**. Overall, NNI demonstrated a direct positive synchronization with the nitrogen application gradients. Under the excessive N3 treatment, NNI remained consistently above the optimal threshold throughout the entire lifespan, varying from 1.04 to 1.22. The conventional N2 treatment closely matched or slightly exceeded the balanced status at most key intervals, remaining bounded within a stable range of 0.95 to 1.15. In sharp contrast, the nitrogen-limited N1 and CK treatments dropped significantly below the optimum balance during the critical middle and late reproductive stages (S4–S8), exhibiting constrained NNI spans of 0.72–0.89 and 0.81–0.95, respectively. This progressive differentiation among treatments became most pronounced after the flowering transition, capturing the highly stage-dependent biological feedback of greenhouse cucumber cultivation to external nutrient inputs.

### 3.2 Hyperspectral response to nitrogen stress and sensitive band selection

Leaf hyperspectral reflectance curves analyzed across distinct cucumber growth stages exhibited typical vegetation signatures, characterized by a green reflectance peak near 550 nm, a chlorophyll absorption valley around 670 nm, a sharp red-edge inflection near 700 nm, and a high-reflectance plateau in the near-infrared (NIR) region spanning 750–1000 nm (**Figure 4**). Although the overall spectral profile remained consistent, the reflectance magnitude varied substantially across growth stages and nitrogen levels. At the seedling stage (S1), the excessive N3 treatment exhibited the highest NIR reflectance (67.61%), outperforming other groups by 0.69%–5.48% (**Figure 4A**). At the flowering stage (S3), a general decline in leaf reflectance occurred across all treatments, with N3 showing a more pronounced drop that suggested accelerated canopy closure driven by high nitrogen inputs (**Figure 4C**). Treatment divergence became highly prominent at the early fruit-bearing stage (S5), where the nitrogen-deficient CK group recorded the highest visible-region reflectance (7.00%) due to diminished pigment concentrations, whereas N3 registered the lowest value (5.06%) (**Figure 4E**). Conversely, in the NIR region at S5, N3 demonstrated the lowest reflectance (48.21%), which was 5.78% lower than that of N1. This inversed NIR pattern persisted through the peak fruit-bearing stage (S6), following the order of CK > N1 > N3 > N2 (**Figure 4F**). By the final harvesting stage (S8), N1 maintained the highest reflectance in both the visible (7.44%) and NIR (53.86%) domains, while N3 stabilized at lower values of 6.28% and 51.55%, respectively (**Figure 4H**). These results demonstrate that nitrogen-induced vegetative changes create highly stage-dependent differences in cucumber leaf spectral properties.

**Figure 9:**
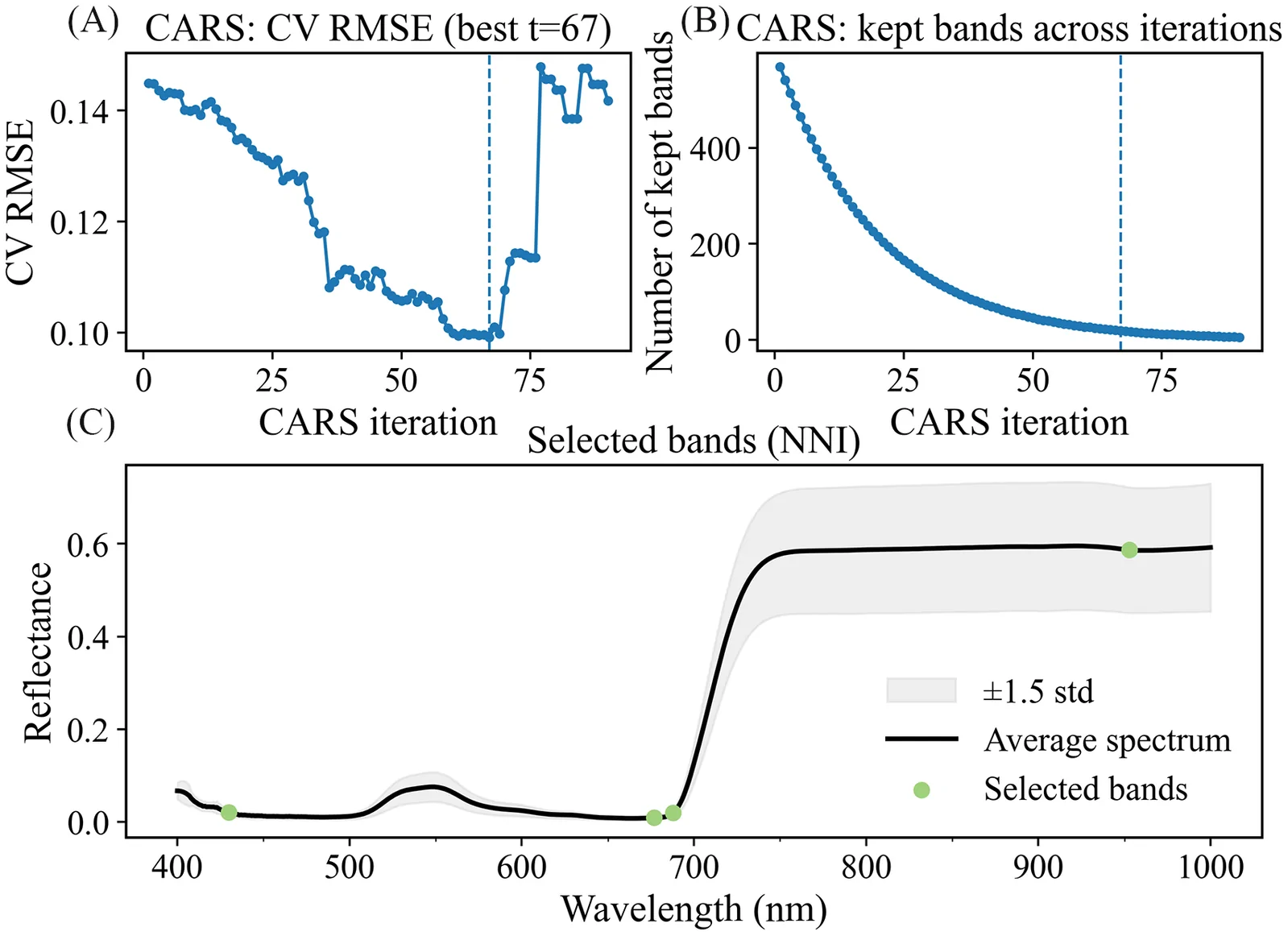
CARS-based selection of NNI-sensitive bands for cucumber nitrogen nutrition index estimation. **(A)** Cross-validation root means square error (CV-RMSE) as a function of iteration number; **(B)** number of retained bands as a function of iteration number; and **(C)** locations of the selected characteristic bands, 430, 677, 688, and 953 nm, on the mean spectral curve.

To overcome full-spectrum data redundancy and mitigate the risk of model overfitting, the CARS–VIF hybrid feature engineering strategy was deployed first to identify the most informative wavebands. During the iterative CARS selection process, the Cross-Validation Root Mean Square Error (CV-RMSE) initially decreased before rising sharply, identifying the 67th iteration as the optimal subset with a minimized CV-RMSE of 0.0991 (**Figure 9A**). Concurrently, the total number of retained wavelengths declined exponentially from the full spectrum (**Figure 9B**). This screening isolated a subset of four characteristic bands: 430, 677, 688, and 953 nm, successfully achieving a 99.81% reduction in spectral dimensionality (**Figure 9C**). These selected wavelengths align precisely with key physiological features: 430 nm resides in the blue chlorophyll *a* and carotenoid absorption zone; 677 nm anchors the red chlorophyll *a* absorption peak; 688 nm targets the highly sensitive rising slope of the red-edge; and 953 nm captures structural leaf water absorption properties. Subsequent VIF collinearity diagnostics confirmed that all four bands remained below the critical threshold (VIF < 10), proving that severe multicollinearity was systematically eliminated. Consequently, this optimized, multi-band, collinearity-free feature set was established as the mathematical input for the subsequent NNI inversion modeling.

**Figure 10:**
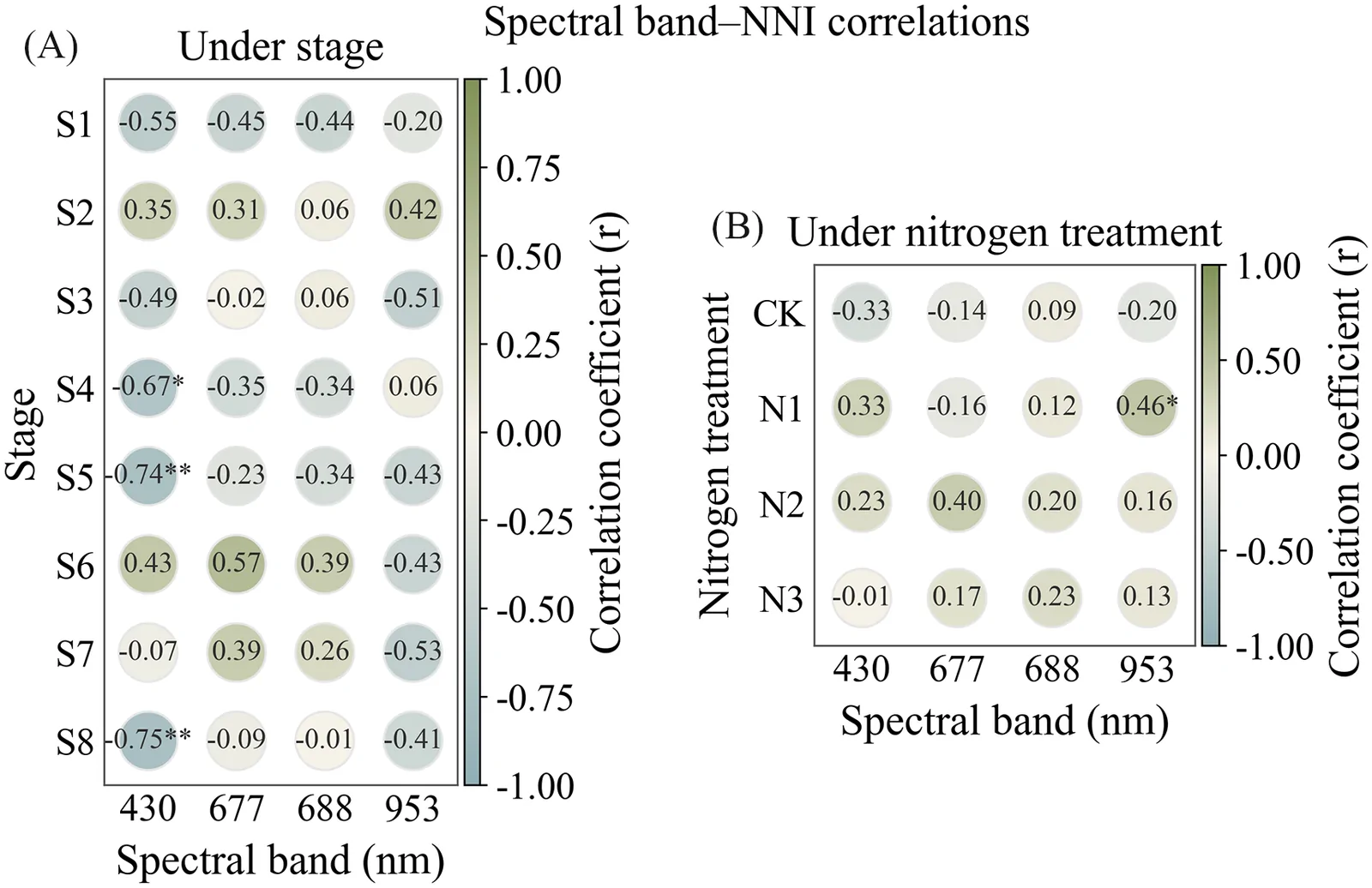
Relationships between spectral reflectance at selected bands and cucumber nitrogen nutrition index (NNI) under different growth stages and nitrogen treatments. Pearson correlation coefficients between NNI and reflectance at the selected bands, 430, 677, 688, and 953 nm, across different growth stages (S1–S8). **(B)** Pearson correlation coefficients between NNI and reflectance at the selected bands under different nitrogen treatments.

Following the feature selection, Pearson correlation coefficients (r) between NNI and reflectance at the four selected wavebands (430, 677, 688, and 953 nm) were calculated across growth stages and nitrogen gradients to decode the underlying relationships between spectral shifts and crop nutrient variations (**Figure 10**). Temporally, the correlations exhibited substantial non-linear and stage-dependent fluctuations (**Figure 10A**). Among the wavelengths, the blue-region 430 nm band exhibited the most robust and consistent negative correlations with NNI, reaching statistical significance during the fruit-setting (S4), early fruit-bearing (S5), and vine-pulling (S8) stages *r* = -0.67 to -0.75, *p* < 0.05). The red (677 nm) and red-edge (688 nm) bands followed a synchronized temporal trajectory, shifting from negative correlations during early vegetative development to positive correlations during the middle-to-late reproductive phases. In contrast, the NIR 953 nm band maintained negative correlations with NNI across most key intervals, displaying stronger correlation values from S3 through S8. Grouped analysis by nitrogen treatments further revealed context-dependent correlation behaviors (**Figure 10B**). Under the CK and N3 boundary treatments, the linkages between NNI and single-band reflectance were generally suppressed. Under the low-nitrogen N1 treatment, however, 953 nm exhibited a significant positive correlation with NNI (*r* = 0.46, *p* < 0.05), followed closely by 430 nm. Conversely, under the conventional N2 level, all selected wavelengths transitioned into positive correlations, with 677 nm emerging as the dominant driver. This high level of variability confirms that the relationship between single-band reflectance and NNI is highly non-linear and background-dependent, justifying the utilization of advanced machine learning pipelines rather than simple linear indices.

### 3.3 Performance benchmarking and validation of machine learning inversion models

**Figure 11:**
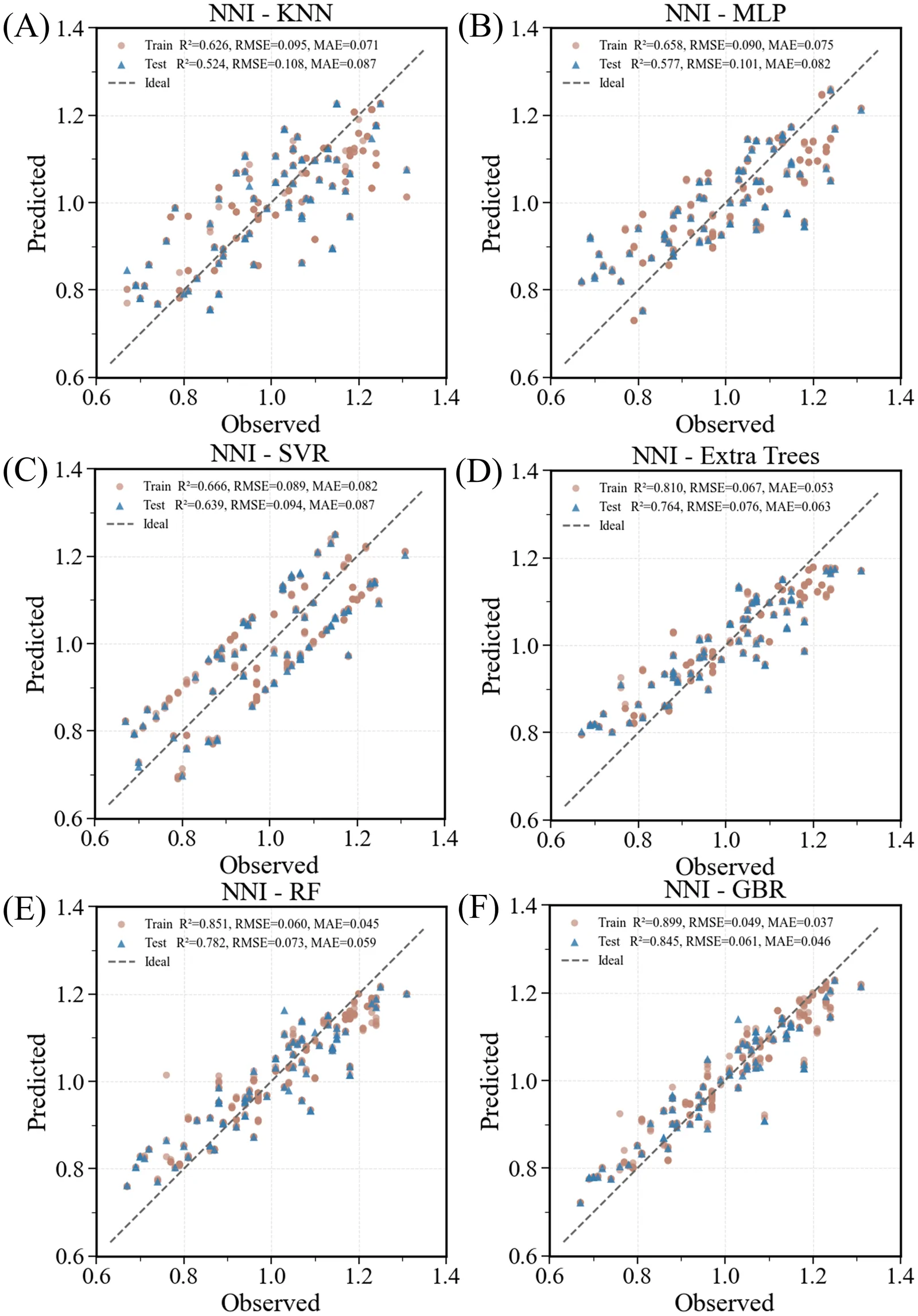
Predicted versus observed nitrogen nutrition index (NNI) values for different machine learning models using the training and test datasets. **(A)** K-Nearest Neighbors (KNN); **(B)** Multilayer Perceptron (MLP); **(C)** Support Vector Regression (SVR); **(D)** Extra Trees; **(E)** Random Forest (RF); and **(F)** Gradient Boosting Regressor (GBR).

Based on the selected multi-band optimal features, six machine learning regression paradigms were deployed to estimate the greenhouse cucumber Nitrogen Nutrition Index (NNI), with their training and independent testing performances systematically benchmarked in **Figure 11**. Predictive accuracy varied substantially among the algorithms, revealing that the ensemble meta-learners fundamentally outperformed the standalone individual mappers. Across ten repetitions of 5-fold cross-validation, Gradient Boosting Regression (GBR) consistently achieved the highest prediction accuracy, delivering a mean R² of 0.842 (± 0.015), a mean RMSE of 0.063 (± 0.004), and a mean MAE of 0.047 (± 0.003). The second-best model, Random Forest (RF), yielded R² = 0.779 (± 0.021), RMSE = 0.074 (± 0.005), and MAE = 0.059 (± 0.004), with non-overlapping standard errors confirming GBR’s statistically superior performance. On the independent test set, GBR achieved R² = 0.845, RMSE = 0.061, and MAE = 0.046 (**Figure 11F**). The other ensemble architectures, including Random Forest (RF) ( $R^{2}=0.782$, RMSE=0.073) and Extra Trees ( $R^{2}=0.764$, RMSE=0.076), also showcased superior predictive capacities relative to most single individual paradigms (**Figures 11D, E**). In sharp contrast, K-Nearest Neighbors (KNN) exhibited the poorest validation capacity, generating a lowest test *R*^2^ of 0.524 (**Figure 11A**). Furthermore, evaluating the performance gap between calibration and validation phases highlighted divergent mathematical behaviors among the models. Support Vector Regression (SVR) demonstrated the most stable generalization with the smallest training–test *R*^2^ gap (0.027) (**Figure 11C**). Conversely, RF and Multilayer Perceptron (MLP) suffered from noticeable generalization decay, with the *R*^2^ of MLP dropping from 0.658 in training to 0.577 in testing (**Figure 11B**). Taken together, GBR provided the most optimal mathematical balance between global fitting precision and test-set generalization stability among all evaluated candidates.

Utilizing this selected GBR inversion pipeline, predicted NNI values were mapped and cross-evaluated against empirical observations across growth stages and nitrogen treatments, demonstrating exceptional spatiotemporal synchronization. The model successfully tracked the positive correlation between nitrogen application rates and NNI magnitudes, consistently mirroring the hierarchy of N3 > N2 > N1 > CK. Grouped developmental analysis by reproductive phases revealed that the GBR-predicted NNI trajectories progressively diverged after the flowering stage (S3), capturing the subtle onset of nutritional differentiation during the high-demand fruit-bearing stages. In contrast to the overlapping, constrained treatment responses observed during the early vegetative window (S1–S2), clear boundaries emerged post S3. The control CK plot remained severely depressed below the 1.0 threshold. The low-nitrogen N1 treatment fluctuated near the lower margin of the optimal range during S3–S5, signaling a latent post-flowering nitrogen limitation before external deficiency traits manifested. Crucially, the conventional N2 rate maintained NNI predictions closest to the theoretical ideal line of 1.0 throughout most reproductive intervals. Conversely, the excessive N3 treatment consistently surpassed 1.05 after S3, indicating a persistent luxurious consumption and major nitrogen surplus. These findings validate that the selected GBR framework provides an accurate, non-destructive, and high-frequency real-time diagnostic toolkit capable of effectively isolating genuine physiological stress and supporting precision fertilization decision-making.

## 4 Discussion

### 4.1 Biological dynamics of cucumber growth and physiological feedback to nitrogen gradients

The foundational milestone of this study is the parameterization of the critical nitrogen ( $N_{c}$) dilution curve ( $N_{c}=4.378\times {\text{DW}}^{-0.115}$, *R*² = 0.745), which serves as a biological benchmark for tracking the dynamic nitrogen status of greenhouse cucumber. The coefficient of determination (*R*² = 0.745) is consistent with the only previously reported cucumber critical N dilution curve under protected cultivation (*R*² = 0.749) (Wang et al., 2025), suggesting that the moderate fit is typical for this crop and experimental context, rather than an indication of poor model performance. The curve parameter *a* (4.378) and *b* (0.115) are also comparable to the published values (*a* = 4.539, *b* = 0.060), with minor differences likely arising from cultivar-specific physiological traits and varying environmental conditions. Dynamically, the structural parameters *a* (4.378) and *b* (0.115) reflect the specific physiological expansion and architectural shading properties of indoor vine crops, distinguishing them from open-field cereal or root crops. Compared to field crops that exhibit sharp biomass accumulation and rapid canopy closure (often resulting in dilution coefficients *b* > 0.400), the relatively conservative dilution exponent (*b* = 0.115) observed here reveals that greenhouse cucumber leaves maintain high vertical nitrogen concentrations throughout their lifespans to maximize continuous photosynthesis under plastic-film diffuse light environments.

Driven by this framework, the calculated empirical NNI (ranging from 0.72 to 1.22) captured clear treatment-specific nutritional boundaries post-flowering (S3). The severe depletion under the CK (0.72–0.89) and N1 (0.81–0.95) treatments during the fruit-bearing stages (S4–S8) highlights a critical physiological bottleneck: the massive sink-strength driven by expanding fruit triggers a rapid remobilization of mobile nitrogen from older vegetative functional leaves to the developing reproductive organs. Conversely, the high NNI plateau (1.04–1.22) observed under the excessive N3 treatment proves that over-fertilization leads to substantial luxurious consumption rather than further biomass optimization, establishing a clear mathematical justification for implementing dynamic diagnostic boundaries.

### 4.2 Optoelectronic mechanics of hyperspectral dimensionality reduction and collinearity elimination

High-dimensional full-spectrum hyperspectral datasets naturally suffer from sever multicollinearity and redundant background noise, which traditionally dampens model generalization. In this study, the deployment of the CARS–VIF hybrid feature engineering strategy effectively addressed this limitation by compressing the extensive spectral matrix down to a collinearity-free subset of four sensitive wavebands (430, 677, 688, and 953 nm), achieving an impressive 99.81% dimensionality reduction. The scientific validity of this compressed subset is strongly backed by the optical and pigment absorption properties of the cucumber leaves. Specifically, 430 nm matches the strong absorption zone of chlorophyll *a* and carotenoids; 677 nm anchors the primary red absorption valley of chlorophyll *a*; 688 nm positions precisely on the highly volatile rising slope of the red edge, which is known to shift towards longer wavelengths under high-nitrogen inputs; and 953 nm captures the structural leaf water status and internal cellular scattering dynamics (Li et al., 2009).

Cross-evaluating the subsequent machine learning regression algorithms confirmed that the ensemble meta-learners successfully exploited these isolated wavebands, fundamentally bypassing the structural limitations of individual mappers. Gradient Boosting Regression (GBR) emerged as the most robust inversion engine, delivering an outstanding test-set *R*^2^ of 0.845, a minimized *RMSE* of 0.061, and the lowest *MAE* of 0.046. The superior performance of GBR over standalone individual architectures like KNN ( $R^{2}=0.524$) or MLP (validation decay from 0.658 to 0.577) is due to its forward-stage-wise additive expansion mechanism. By sequentially fitting weak regression tree base learners to minimize custom empirical loss functions, GBR specifically focuses on the difficult-to-fit spectral residuals, making it highly adept at handling the complex, non-linear relationships between spectral signatures and crop NNI under small-sample constraints (*n* = 384).

### 4.3 Agronomic implications of machine learning diagnoses for precision nutrient management

From a practical agronomic viewpoint, translating the non-destructive GBR–NNI inversion pipeline into greenhouse management offers a powerful technical toolkit to replace the destructive, time-consuming chemical analysis methods traditionally used (Abbott et al., 2023). Our empirical validation tracked the spatiotemporal synchronization of NNI under distinct treatment gradients, proving that the conventional N2 fertilization rate (480 $\text{kg}\cdot {\text{ha}}^{-1}$) maintained the NNI predictions closest to the theoretical ideal line of 1.0 during the high-demand reproductive phases. In contrast, the excessive N3 treatment (720 $\text{kg}\cdot {\text{ha}}^{-1}$) resulted in an NNI surplus consistently exceeding 1.05 after the flowering stage (S3), confirming that modern greenhouse vegetable production is severely compromised by over-fertilization.

Implementing this non-destructive real-time diagnostic pipeline can generate substantial economic and environmental dividends. Politically, this data-driven framework supports China’s national green development directives, specifically the “Chemical Fertilizer Zero-Growth Action Plan.” By replacing regional, uniform top-dressing strategies with high-frequency, stage-dependent nitrogen management driven by the GBR model, commercial growers could potentially reduce chemical nitrogen inputs (e.g., transitioning from the excessive N3 level to the conventional N2 level), as our NNI diagnostics clearly indicate that the N3 treatment resulted in sustained nitrogen surplus throughout the reproductive stages. However, we acknowledge that yield response data are required to confirm the agronomic and economic outcomes of such reductions. Nevertheless, this targeted fertilization approach is expected to directly mitigate the severe agricultural non-point source pollution risks—such as nitrate leaching into groundwater and secondary soil salinization—that currently compromise intensive greenhouse vegetable production areas in northern China.

### 4.4 Uncertainties in model transferability and cross-scale extrapolation horizons

Despite the outstanding accuracy achieved by the optimized CARS–VIF–GBR modeling framework, several inherent limitations must be addressed before wide-scale commercial application. First, the experimental datasets were gathered using a single summer cucumber cultivar within a localized greenhouse microclimate in Tianjin, China. Because the regression architectures of machine learning algorithms are highly dependent on their training environments, the optimized model parameters might exhibit structural instability if applied to winter cultivation seasons or alternative cultivars with divergent leaf canopy structures. Second, while transferring selected functional leaves to the laboratory secured highly stable spectral curves by eliminating ambient light variations, this destructive sampling step still limits true, high-throughput automated field diagnostics. Furthermore, we acknowledge that the current dataset was derived from a single cucumber cultivar (‘Jinyou 35’), cultivated in a single greenhouse located in Tianjin, China, during one summer growing season. The specificity of this experimental design necessarily constrains the generalizability of our findings. Machine learning models are known to be sensitive to training data distributions; thus, the optimal wavebands and GBR model parameters identified in this study may not directly transfer to other cultivars with distinct canopy architectures, different greenhouse microclimates, or alternative growing seasons (e.g., winter with lower light intensity and temperature). Future work should incorporate multi-cultivar, multi-site, and multi-season experimental matrices to systematically evaluate and enhance the robustness and transferability of the proposed NNI inversion framework across the full spectrum of commercial production environments.

Future research should focus on overcoming these regional and architectural boundaries. To elevate model transferability and generalization ceilings, multi-year, multi-site experimental matrices encompassing distinct greenhouse architectural styles, growing seasons, and diverse vegetable species should be integrated into the cloud training data bank. Methodologically, future efforts should focus on transferring this leaf-level feature subset onto Unmanned Aerial Vehicle (UAV) or automated robotic sensor platforms equipped with low-cost multispectral cameras targeted at the 430, 677, 688, and 953 nm channels (Ali et al., 2024). This scaling transition from microscopic leaf sampling to macroscopic canopy monitoring will accelerate the deployment of high-frequency, non-destructive, and fully automated precision nitrogen diagnostic networks in modern smart facility agriculture. Additionally, while repeated cross-validation was employed to strengthen the statistical confidence of model comparisons, the calibration dataset was derived from a single experimental season. Future work should incorporate multi-season and multi-location data to further enhance model generalization and robustness. Furthermore, while we performed CARS-VIF feature selection exclusively on the training set to prevent test set information leakage, we acknowledge that embedding feature selection within the cross-validation loop would provide an even more rigorous safeguard against overfitting. This represents a methodological refinement to be considered in future studies with expanded datasets. We acknowledge that this study employed default hyperparameters for all machine learning models to ensure fair algorithm comparison, and no systematic hyperparameter tuning was conducted. While CARS-VIF was used for feature selection and six models were compared for algorithm selection, future work should incorporate hyperparameter optimization (e.g., grid search or Bayesian optimization with cross-validation) to further improve model performance. The use of default parameters represents a methodological simplification that may not fully exploit the predictive potential of each algorithm.

## 5 Conclusion

In this study, a robust and non-destructive machine learning-driven inversion framework was successfully established to diagnose the nitrogen nutritional status of greenhouse cucumber. Based on synchronized plant aboveground biomass and plant nitrogen concentration datasets, the critical nitrogen dilution curve for greenhouse cucumber was accurately parameterized as $N_{c}=4.378\times {\text{DW}}^{-0.115}$( $R^{2}=0.745$, p<0.001), delineating rigorous physiological thresholds to calculate the empirical Nitrogen Nutrition Index (NNI). To eliminate full-spectrum data redundancy and severe multi-band multicollinearity, the CARS–VIF hybrid feature engineering strategy effectively compressed the extensive spectral matrix down to a collinearity-free subset of four sensitive wavebands (430, 677, 688, and 953 nm), achieving a 99.81% reduction in spectral dimensionality.

Among the six bench-marked regression models, Gradient Boosting Regression (GBR) delivered the most optimal mathematical balance between global fitting precision and test-set generalization stability, achieving an outstanding validation *R*^2^ of 0.845, a minimized *RMSE* of 0.061, and the lowest *MAE* of 0.046. The GBR-predicted NNI trajectories captured clear treatment-specific nutritional boundaries post-flowering, successfully isolating genuine physiological nitrogen deficiency from surplus conditions. Taken together, these findings demonstrate that coupling leaf hyperspectral technology with optimized ensemble meta-learners enables high-frequency, non-destructive, and reliable diagnosis of crop nutrient status for the specific conditions tested in this study, providing a methodological foundation for precision nitrogen management in greenhouse cucumber production. Future work will focus on evaluating the transferability of this framework across diverse cultivars, growing seasons, and greenhouse environments, and on translating this leaf-level approach to UAV-based canopy-level monitoring for operational deployment.

## Data Availability

The raw data supporting the conclusions of this article will be made available by the authors, without undue reservation.
